# Effect of *NK4* Transduction in Bone Marrow-Derived Mesenchymal Stem Cells on Biological Characteristics of Pancreatic Cancer Cells

**DOI:** 10.3390/ijms15033729

**Published:** 2014-03-03

**Authors:** Yun-Peng Sun, Ben-Long Zhang, Jian-Wen Duan, Huan-Huan Wu, Ben-Quan Wang, Zheng-Ping Yu, Wen-Jun Yang, Yun-Feng Shan, Meng-Tao Zhou, Qi-Yu Zhang

**Affiliations:** 1 Department of Hepatobiliary Surgery, the First Affiliated Hospital of Wenzhou Medical University, Wenzhou 325015, Zhejiang, China; E-Mails: sunyunpeng0123@126.com (Y.-P.S.); djw3522@163.com (J.-W.D.); wangbeq@sohu.com (B.-Q.W.); yjping45@tom.com (Z.-P.Y.); greatywj@sohu.com (W.-J.Y.); syfsjb@sina.com (Y.-F.S.); zmtzmtao@126.com (M.-T.Z.); 2 Department of General Surgery, Yiwu Chouzhou Hospital, Yiwu 322000, Zhejiang, China; E-Mail: zhangbenlong0021@126.com; 3 Department of Infectious Disease, the First Affiliated Hospital of Wenzhou Medical University, Wenzhou 325015, Zhejiang, China; E-Mail: wuhh19@sina.com

**Keywords:** *NK4*, pancreatic cancer, bone marrow-derived mesenchymal stem cells, transduction

## Abstract

Pancreatic cancer usually has a poor prognosis, and no gene therapy has yet been developed that is effective to treat it. Since a unique characteristic of bone marrow-derived mesenchymal stem cells (MSCs) is that they migrate to tumor tissues, we wanted to determine whether MSCs could serve as a vehicle of gene therapy for targeting pancreatic cancer. First, we successfully extracted MSCs from SD rats. Next, MSCs were efficiently transduced with *NK4*, an antagonist of hepatocyte growth factor (HGF) which comprising the *N*-terminal and the subsequent four kringle domains of HGF, by an adenoviral vector. Then, we confirmed that rat MSCs preferentially migrate to pancreatic cancer cells. Last, MSCs expressing *NK4* (*NK4*-MSCs) strongly inhibited proliferation and migration of the pancreatic cancer cell line SW1990 after co-culture. These results indicate that MSCs can serve as a vehicle of gene therapy for targeting pancreatic cancer.

## 1. Introduction

Pancreatic cancer is a very aggressive malignant tumor, and has an extremely poor prognosis [1,2]. Since most pancreatic cancers are diagnosed late and often have invaded surrounding tissues, local treatment such as surgery of advanced pancreatic cancer is not a viable option. Although some adjuvant therapies, such as intraoperative radiotherapy and adjuvant chemotherapy have been tested, satisfactory results have not been obtained as of yet [3]. Recent advances in our understanding of the genetics and epigenetics of pancreatic cancer have revealed that alterations in several tumor-related genes, including *K-ras*, *p53*, matrix metalloproteinases (*MMP*), hepatocyte growth factor (*HGF*), and epidermal growth factor receptor [4–6], may underlie the aggressiveness of this neoplasm [7]. Gene therapy therefore provides a promising new approach for treating this often fatal disease.

Human mesenchymal stem cells (MSCs) derived from bone marrow (BM) comprise an adult population that may participate in the repair of tissue damage inflicted by natural expiry of cells, injury or disease [8]. MSCs are thought to contribute to the regeneration of mesenchymal tissues such as bone, cartilage, muscle, ligament, tendon, adipose tissue and stroma. The ability of MSCs to migrate to areas of injury and to tumors has encouraged investigation of MSCs as therapeutic tools. MSCs have also been used for targeted delivery of therapeutic gene products to the tumor microenvironment in animal models [9,10], and the therapeutic use of MSCs is being explored for various disease conditions. Recent advances in studying MSC biology have shown that this cell population exhibits some properties that suggest the feasibility of their use as a cellular vehicle: a relative simple isolation method, the ability to be cultured *in vitro* and to expand to quantities required for therapy, the ability for *ex vivo* transduction with viral vectors, plasticity, the potential to differentiate under exogenous stimuli, high metabolic activity, an efficient machinery to express therapeutic proteins and the ability to be delivered systemically or locally [11].

Hepatocyte growth factor (HGF), originally identified as a mitogen of mature hepatocytes with a 69 kDa α-chain and 34 kDa β-chain, is a multifunctional growth factor that stimulates mitogenesis, motogenesis and morphogenesis in a variety of epithelial and endothelial cells [12,13]. HGF is also a strong inducer of tumor growth, angiogenesis and lymphangiogenesis, thereby promoting invasion and metastasis of tumor cells [14–16]. The biological responses to HGF are mediated through its receptor, c-Met, a receptor tyrosine kinase expressed in a variety of cells. Because c-Met is inappropriately expressed in almost all types of human cancer, the HGF/c-Met signaling pathway should be an attractive target for cancer therapies [17]. The HGF-antagonist-designated *NK4*, consisting of the NH_2_-terminal hairpin domain and the four kringle domains of the HGF α-chain, binds to c-Met and competitively antagonizes HGF-induced tyrosine phosphorylation of c-Met, resulting in inhibition of HGF-mediated cell proliferation, migration, invasion, angiogenesis and lymphangiogenesis and in antiapoptotic effects that contribute to malignant growth of tumor cells [18]. *NK4* had been reported to inhibit cell proliferation by transfer to pancreatic cancer cells [19]. However, the systemic administration of viral vectors often causes severe adverse effects, such as liver toxicity and humoral and cytotoxic immune responses.

In this study, we introduced the *NK4* gene into MSCs by using an adenoviral vector and investigated it affects on the proliferation, apoptosis and migration of pancreatic cancer cells. The objective of this study was to explore whether BM-MSCs may be used as a *NK4* carrier for human pancreatic cancer treatment––a possibility that would require an experimental basis for the clinical application of manipulations of the *NK4* gene product.

## 2. Results and Discussion

### 2.1. Isolation and Character Identification of Bone Marrow-Derived MSCs

**Figure 1.**
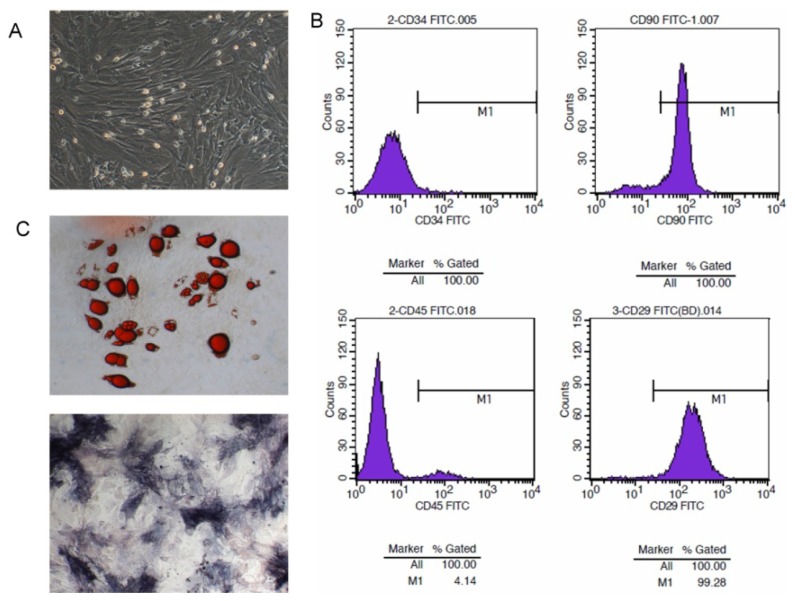
Morphology and characteristics of bone marrow-derived MSCs. (**A**) MSC development and morphology was observed with Olympus microscopy (Olympus Corporation, Tokyo, Japan); (**B**) Immunophenotypic profile of MSCs. Flow cytometric histograms indicated the positive expression of CD29, CD90, while cells were negative for CD34 and CD45; (**C**) Adipogenic and osteogenic differentiation abilities of MSCs. Lipid droplets were visualized by staining with Oil Red O solution. Alkaline phosphatase granules located in the cytoplasm stained intensively with a darker color than the cell.

MSCs were isolated from murine bone marrow cells after passage three or four generations, when they displayed a large and polygonal morphology (Figure 1A). In order to verify the character of bone marrow-derived MSCs, they were analyzed by flow cytometry for the expression of surface markers. These cells revealed the typical antigenic profile of MSCs and were positive for CD29 and CD90 antigens as reported previously [20,21]. In contrast, these cells were negative for CD45 and CD34 (Figure 1B); since CD45 [22,23] and CD34 [24] are positive markers for hematopoietic stem cells, (HSC) differentiation from MSCs though CD34 is currently controversial [25]. Furthermore, induced directional differentiation tests *in vitro* showed that adipose cells and osteocytes were stained after MSC induction through lipoblast and osteoblast inducers (Figure 1C). The MSCs had a differentiation capability for adipogenesis and osteogenesis.

### 2.2. Recombinant Adenoviral-Mediated Gene Delivery to HEK293 Cells

**Figure 2.**
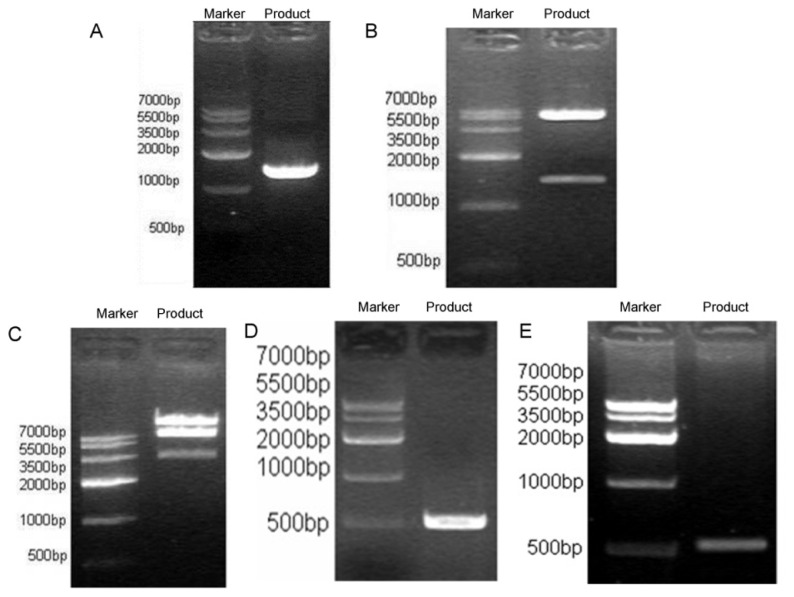
Recombinant adenoviral-mediated gene delivery to HEK293 cells. (**A**) The *NK4* gene fragment was amplified by PCR (about 1.45 kb) by using the plasmid pBluescript II-SK(+)-HGF as template, the amplified products were analyzed and detected using the GIS gel system; (**B**) The *NK4* gene fragment was sub-cloned into pYr-adshuttle-6 carrier. Positive clones identified by NheI and SalI double digestion can produce 1.45 kb fragment band and 5.8 kb carrier band, speculating that *NK4* gene fragment has been cloned into pYr-adshuttle-6; (**C**) The *NK4* gene fragment (*NK4*-IRES-EGFP) *in vitro* was cloned into adenovirus expression vector pAd/BL-DEST using recombinant LR technology. Positive clones can produce small, *ca.* 3.5, 7.5 kb fragments as well as greater than 15 kb fragments, indicating that the *NK4* gene fragment has been cloned into pAd/BL-DEST vector, named pAd-*NK4*; (**D**) The *NK4* fragment was amplified by PCR (about 500 bp) by using the plasmid pAd-*NK4* as template, the amplified products were analyzed and detected in the GIS gel system, and the consistency of the two fragments confirmed that the pAd-*NK4* vector was successfully constructed; (**E**) PCR analysis for *NK4* confirmed proper recombination.

Amplification of *NK4* cDNA was then performed using specific primers and the pBluescript II-SK(+)-HGF plasmid as template, which resulted in a single band of about 1434 bp on agarose gel electrophoresis (Figure 2A). The PCR product is directionally cloned into pYr-adshuttle-6, which is a shuttle vector for Gateway recombination cloning (Figure 2B). Then, the fidelity of the cloned sequence was confirmed by sequencing. Next, the LR recombination reaction which usually performed for transferring vector sequence to one or more carriers was performed between the clone (pYr-adshuttle-6-*NK4*) (Figure 2C) and the destination vector (pAd/BL-DEST) (Figure 2D) to form pAd-*NK4*. Finally, the HEK293 cells were transfected with PacI linearized recombinant adenovirus pAd-*NK4* to form recombinant adenovirus rAd-*NK4*/EGFP, and proper recombination was confirmed by PCR analysis using gene specific primers for *NK4* (Figure 2E).

### 2.3. Transient Expression of *NK4* in MSCs Using Adenoviral Vector

**Figure 3.**
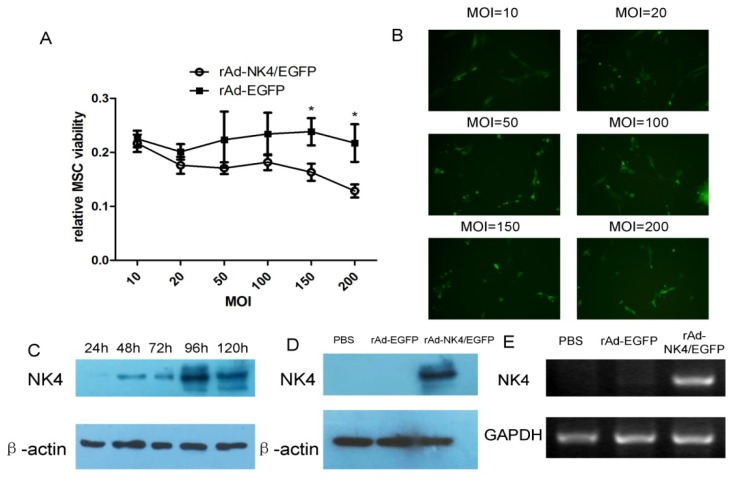
*NK4* transfection expression in MSCs using adenoviral vectors. (**A**) Relative MSCs viability with increasing MOI between rAd-*NK4*/EGFP and rAd-EGFP. * *p* < 0.05. The data are presented as mean ± SD of triplicate samples; (**B**) Transfection of MSCs with increasing concentrations of rAd-*NK4*/EGFP (10, 20, 50, 100, 150 and 200 MOI). The optimization of transfection efficiency was decided by the images of immunofluorescence of EGFP; (**C**) The expression of *NK4* in different times when the MSCs were transfected at MOI = 100. After transfection for 96 h, *NK4* expression was highest compared with other time points; (**D**) *NK4* expression for rAd-*NK4*/EGFP transfected MSCs with optimal dose and time compared with the rAd-EGFP and the control groups with PBS; (**E**) *NK4* expression in MSCs verified by RT-PCR. MSCs were tranduced with rAd-*NK4*/EGFP, rAd-EGFP or PBS, respectively.

After incubation for 24 h with monolayer cells up to 80% confluence, the fourth passage of the MSCs was infected with rAd-*NK4*/EGFP at different levels of multiplicity of infection (MOI). The transfection rate was similar under different MOI (data not shown). 3-(4,5-Dimethylthiazol-2-yl)-2,5- diphenyltetrazolium bromide (MTT) assay was used to compare the differences in the viability of MSCs. The viability of MSCs in groups of MOI = 150 and MOI = 200, showed significant difference between rAd-*NK4*/EGFP and rAd-EGFP groups (Figure 3A); the optimization of transfection efficiency was decided by the expression of immunofluorescence of EGFP in the images (Figure 3B). From these results, MOI = 100 was chosen as the appropriate MOI.

The fourth passage of the MSCs was infected with appropriate multiplicity of infection of the recombinant adenovirus vector harboring the *NK4* (rAd-*NK4*/EGFP) coding sequence or the control viral vector (rAd-EGFP). Western blots were used to compare changes in the expression of *NK4* at different time points (1, 2, 3, 4 and 5 days post-infection). Initial expression of *NK4* was detected on day two and increased to the highest expression level at day four, and then decreased thereafter (Figure 3C).

Accordingly, compared with the control group, the high expression level of *NK4* was also detected by western blotting (Figure 3D) and RT-PCR (Figure 3E) at day 4. These results indicated the transient overexpression of *NK4*. Most of the transduced MSCs continuously adhered to the tissue culture dishes, and no distinct morphological changes were observed.

### 2.4. *NK4* Expression in MSC Cell Supernatant and Oncotropism to Pancreatic Cancer Cells

**Figure 4.**
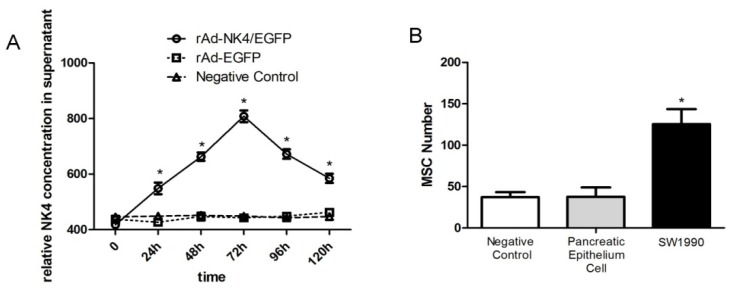
*NK4* expression in MSCs cell supernatant and oncotropism to pancreatic cancer cell. (**A**) MSCs were infected with rAd-*NK4*/EGFP or rAd-EGFP (MOI = 100). Culture supernatants were collected at different times and concentrations of *NK4* in the media were determined through ELISA. *NK4* concentration in the rAd-*NK4*/EGFP group was significantly higher at each time point and peaked at 72 h. The data are presented as mean ± SD of triplicate samples. * *p* < 0.05 SW1990 group compared with the pancreatic epithelium cell group and control group; (**B**) The ability of MSCs to migrate to pancreatic cancer was determined by transwell assays. MSCs were transfected with rAd-*NK4*/EGFP in the upper chamber; pancreatic cancer cell SW1990, pancreatic epithelium cell or PBS were in the lower chamber. Ten visual fields of each chamber were collected for counting. The data are presented as mean ± SD of triplicate samples. * *p* < 0.05 SW1990 group compared with the pancreatic epithelium cell group and control group.

To confirm the whether *NK4* could be secreted to the extracellular environment with stable expression after transfection with rAd-*NK4*/EGFP, ELISA assays were used to detect *NK4* expression levels in MSCs cell supernatant. Over time, *NK4* expression levels increased after MSCs transfection with rAd-NK4/EGFP having the highest level at 72 h then decreasing; the rAd-EGFP group and control group, had no *NK4* expression in the MSCs cell supernatant (Figure 4A). This indicated that *NK4* protein could be expressed in the MSCs and secreted out of the MSCs.

Transwell migration assays wereused to test the oncotropism of MSCs to pancreatic cancer cells. After 24 h, the number of rAd-*NK4*/EGFP MSCs migrated to the contralateral of the membrane was considerably larger in the pancreatic cancer cell group than in the pancreatic epithelial cell group and control (Figure 4B). There was no significant difference between the pancreatic epithelial cell group and control. This indicated rAd-*NK4*/EGFP MSCs have obvious tropism to pancreatic cancer *in vitro* and no distinct tendency to pancreatic epithelial cells.

### 2.5. Effect of Transfected MSCs on Biological Characteristics of Pancreatic Cancer Cell

**Figure 5.**
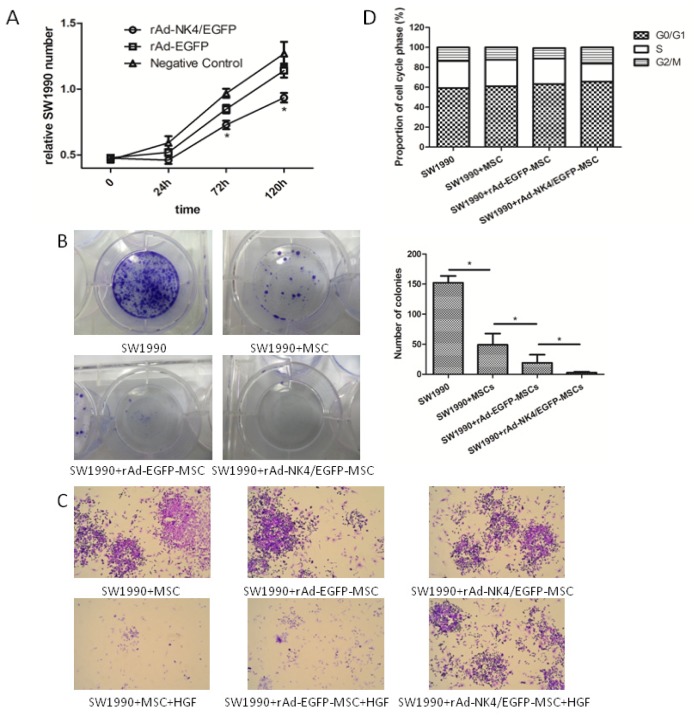
Effect of transfected MSCs on the biological characteristics of pancreatic cancer cells. (**A**) Effect of transfected MSCs on SW1990 cell proliferation. Fewer SW1990 cells grew in the rAd-*NK4*/EGFP MSCs group on the fifth day compared with the rAd-EGFP MSCs group and control. Cell proliferation of different groups was evaluated by MTT assay. * *p* < 0.05. The data are presented as mean ± SD of six samples; (**B**) Effect of transfected MSCs on SW1990 cell colony formation and colony-forming ability of rAd-*NK4*/EGFP MSCs on SW1990 cells. SW1990 cells, MSCs, rAd-EGFP MSCs and rAd-*NK4*/EGFP MSCs were grown in 1640 Medium for 21 days and fed with media every two days. The colonies formed in the medium were stained with 5% Giemsa and observed. Magnified views of selected colonies are shown on the bottom panel. Number of colonies grown in medium were counted under the light microscope and plotted. Bars represent standard deviation (*n* = 3, * *p* < 0.05); (**C**) Effect of transfected MSCs on SW1990 cell scatter. Cells were treated for 48 h by HGF/SF (10 μg/L) to induce cell scattering. rAd-*NK4*/EGFP MSCs inhibit SW1990 cells scatter significantly after 48 h compared with the control group. The SW1990 cells were stained as purple while the MSCs were stained as black; (**D**) Effect of transfected MSCs on SW1990 cell cycle. Flow cytometric analysis was used to determine the alterations in the cell cycle distributions in SW1990 cell lines following the administration of MSCs, rAd-EGFP MSCs and rAd-*NK4*/EGFP MSCs. The data are presented as mean ± SD of triplicate samples. * *p* < 0.05 rAd-*NK4*/EGFP group compared with the rAd-EGFP group and control group.

MTT assays were performed to investigate to effect of rAd-*NK4*/EGFP MSCs on the proliferation of pancreatic cancer cells *in vitro*. MSCs, rAd-EGFP MSCs and rAd-*NK4*/EGFP MSCs were co-cultured with pancreatic cancer cell line SW1990 to study the effect of MSCs secreted *NK4* on cancer proliferation. As shown in Figure 5A, the growth rate of SW1990 cells was slower than in the control groups during the early stages of tumor formation which was consistent with the previous finding that *NK4* is required for the growth of the cancer [26–28]. This result indicated that SW1990 cells proliferation is significantly inhibited by rAd-*NK4*/EGFP MSCs co-culture.

To measure the effect of MSCs on pancreatic cancer cell viability, colony-forming assay was used. SW1990 were seeded, as described in Methods, in dishes after co-culture with different MSCs groups for 72 h (Figure 5B). The absolute number of colony forming cells in the rAd-*NK4*/EGFP MSCs group was significantly decreased at 3 weeks after co-culture, in comparison to controls. Though this indicated that MSCs induced a partial inhibition of colony formation in the control group, the ability of SW1990 to form large colonies was completely inhibited by rAd-*NK4*/EGFP MSCs.

To evaluate the characteristic changes of migration in pancreatic cancer cells co-cultured with MSCs, cell scatter assay was applied. Briefly, SW1990 cells were allowed to grow together with MSCs and treated with HGF to induce scattering. A colony is judged as “scattered” when half of the cells have lost contact with their neighbors and exhibited a fibroblast-like morphology. As shown in Figure 5C, an obvious scattering of SW1990 cells was induced following 48 h treatment with HGF and control MSCs, while in the rAd-*NK4*/EGFP MSC treated group the phenomena of HGF-induced cell scatter was inhibited.

To further evaluate the effect of rAd-*NK4*/EGFP MSCs treatment upon cell cycle profiles, flow cytometry was performed. The data showed that rAd-*NK4*/EGFP MSCs increased the proportion of SW1990 cells in the G1 phase from 59.3% to 65.6%, (Figure 5D) and decreased that in the S phase from 27.1% to 18.1%, indicating that rAd-*NK4*/EGFP MSCs inhibit cell cycle progression. This result suggests that rAd-*NK4*/EGFP MSCs could block the SW1990 cell cycle progression by inhibiting the G1-S phase transition and arresting cells in G1.

### 2.6. Discussion

In this study, we demonstrated that systemically administered MSCs expressing *NK4* efficiently migrated to the pancreatic cancer cells and inhibited tumor progression. This antitumor effect was due to the suppression of epithelial-mesenchymal transition (EMT) and cell cycle in the cancer cell. We also confirmed higher gene transduction efficiency of MSCs with an adenovirus vector using the Gateway technique, compared to conventional adenoviral vectors.

We found that systemically administered MSCs migrated efficiently to cancer cells in the pancreas but not to normal cells. These findings are consistent with earlier reports that systemically administered MSCs can migrate to tumors but not to healthy tissues [9,10,29,30]. Recent reports have shown that bone marrow-derived MSCs are able to recognize the sites of tumors and wounds and contribute to the development of solid tumors and the healing of wounds by differentiating into appropriate cells [31,32]. Although the molecular processes that drive migration of MSCs to tumor tissues remain unclear, the tropism of MSCs to tumor tissues could be mediated, at least in part, by specific growth factors and chemokines [33,34].

Successful treatment of multiple tumors by engineered MSCs has been reported. Intravenous injection of human MSCs expressing interferon-β into SCID mice successfully treated multiple lung metastases by inhibiting tumor growth in the lung [9]. However, high titers (3000 MOI) of the Ad5 vector were needed for the expression of appropriate amounts of interferon-β because of the low efficiency of gene transduction to MSCs. In contrast, an adenoviral vector with Gateway technique could efficiently transduce the genes to MSCs at a relatively low dose (100 MOI).

Malignant tumor cell proliferation is an important characteristic of cancer development, and EMT has an important role in the promotion of oncogenesis, tumor development and growth. In our study, MSCs secreting *NK4* inhibited proliferation of SW1990 cells *in vitro* in a short period. Over a longer time, MSCs secreting *NK4* significantly inhibited SW1990 cell viability, while MSCs alone may also partially have a similar result. This is an interesting phenomenon which may be caused by the different source of MSCs and SW1990 cells, and need further study. Previously, it was reported that injection of an adenoviral vector expressing *NK4* into tumors strongly inhibited cancer cell growth in human pancreatic cancer [35]. The ability of *NK4* to inhibit lung tumor angiogenesis and lymphangiogenesis and induction of apoptosis had also been reported [36]. All of this indicates the significant impact of *NK4* in tumor inhibition and the possibility of MSC to deliver *NK4* to the neoplastic foci. Therefore, MSCs expressing *NK4* is worth further study in pancreatic cancer animal models and may have potential value in clinical therapy of pancreatic cancer.

The mechanism by which *NK4* inhibits proliferation in SW1990 cells may be its inhibitory effect on HGF-induced EMT. Death from cancer is generally associated with metastasis; a biological phenomenon linked to the tumor cell’s ability to migrate, seed and colonize distant sites. Hepatocyte growth factor has attracted considerable attention as a stromal-derived mediator in tumor-stromal interaction, on the basis of its paracrine action and close involvement in cancer growth, invasion and metastasis. As a competitive antagonist of HGF, *NK4* binds to the c-Met receptor, without eliciting any biological influence, thus preventing HGF from activating the receptor [37]. Through our *in vitro* assay, we found that MSCs secreting *NK4* could inhibit HGF-induced cell scatter. Cell scattering is used to describe the dispersion of compact colonies of epithelial cells induced by certain soluble factors. Because the scattering of epithelial colonies possesses characteristics of epithelial-mesenchymal transition, such as the loss of epithelial cell-cell junctions and the acquisition of a motile mesenchymal cell phenotype, the scatter assay has been used for studying epithelial-mesenchymal transition and for detecting factors able to induce migratory behavior of cells. Many reports had proved HGF can induce EMT through the PI3K/Akt/mTOR signaling pathway in several types of cancers [38,39]. This may also play an important role in pancreatic cancer and be worth future study.

Since many factors including the cell cycle can impact the proliferation of the tumor, we investigated the SW1990 cell cycle stage after treatement with MSCs expressing *NK4*. As shown in Figure 5D, MSCs secreting *NK4* arrested the SW1990 cell cycle by decreasing the number of cells in S-phase and increasing those in the G1-phase. The results of recent studies showed that the PI3K/Akt signaling pathway regulates fundamental cellular functions, such as transcription, translation, proliferation, growth, and survival [40]. PI3K activity is important for the G1-to-S transition [41], and Akt regulates a platelet derived growth factor-induced decrease in p27kip1, a nuclear protein that inhibits both the G1- and S-phases [42]. PI3K/Akt activation and subsequent inhibitory phosphorylation of GSK-3β is required for HGF-induced suppression of RANTES in human kidney proximal tubule epithelial cells [43]. Furthermore, the PI3K/Akt signaling pathway is a major regulator of GSK-3β, an important component of diverse signaling pathways involved in the regulation of cell fate, protein synthesis, glycogen metabolism, cell mobility, proliferation, and survival [44]. The SW1990 cell cycle arrest caused by MSCs secreting *NK4* may play its role through blocking the PI3K/Akt/GSK-3β pathway; the exact mechanism needs further investigation.

Taken together, these results indicate that MSCs can serve as a vehicle of gene therapy for targeting pancreatic cancer.

## 3. Experimental Section

### 3.1. Rat and Cells

Eight- to ten-week-old SD rats were purchased from the laboratory animal center of Wenzhou Medical University. All experiments were performed under protocols approved by the Institutional Review Board of animal experiments of Wenzhou Medical University. Human embryonic kidney (HEK) 293 cells were purchased from the American Type Culture Collection (ATCC, Manassas, VA, USA). Pancreatic cancer cells SW1990 and pancreatic epithelial cells were provided from the Shanghai cell bank, Chinese Academy of medical science.

### 3.2. Preparation and Culture of Bone Marrow-Derived MSCs

MSCs were isolated from bone marrow cells by adherence purification on tissue culture plastic. Briefly, bone marrow cells of 8- to 10-week-old SD rats were obtained by flushing the femora and tibiae with Dulbecco’s modified Eagle’s medium-low glucose (DMEM; Gibco, Grand Island, NY, USA). The cells were collected and seeded at a density of 3 × 10^5^ cells/cm^2^ in 24-well plates and grown in DMEM supplemented with 10% fetal bovine serum (FBS; Gibco, Life te, Grand Island, NY, USA), 50 U/mL penicillin and 50 mg/mL streptomycin for 72 h followed by re-feeding of fresh media. When the culture reached confluence, the plastic-adherent cells were lifted up with 0.25% trypsin and 1 mM EDTA, suspended in fresh media and transferred into a new flask. Finally, large and polygonal cells (MSCs) were collected and used during the third or fourth passage for experiments.

### 3.3. Molecular Characterization of MSC

Immunophenotypic surface marker analysis was performed on a FACS Canto II (BD, Heidelberg, Germany) upon staining with the following antibodies as described before: CD29-phycoerythrin (PE, clone MAR4; BD), CD34-APC (clone 8G12; BD), CD45-APC (clone HI30; BD), CD90-APC (clone 5E10; BD).

For adipogenic differentiation, MSC were cultured in medium supplemented with 1 mM dexamethasone, 10 mg/L insulin, 50 mM IBMX, and 0.2 mM indometacin. Twenty days after induction, cells were fixed with 40 g/L paraformaldehyde and lipid droplets were visualized by staining with Oil Red O solution [45]. Fluorescence microscopic pictures were taken from four randomly chosen areas with a Leica DM IL HC microscope (Leica, Wetzlar, Germany).

Osteogenic differentiation was induced with culture medium supplemented with 1 mM dexamethasone, 1 M β-sodium glycerophosphate, and 50 mM ascorbic acid and with medium changes every 3–4 days. After 40 days, osteogenic differentiation was analyzed by alkaline phosphatase staining. Alkaline phosphatase granules located in the cytoplasm stained intensively and the color was darker than the cell. Fluorescence microscopic pictures were taken from four randomly chosen areas with a Leica DM IL HC microscope (Leica Microsystems, Wetzlar, Germany).

### 3.4. *NK4* Gene Cloning to Adenovirus Shuttle Vector pYr-Adshuttle-6

Template plasmid pBluescript II-SK(+)-HGF DNA was sequencing and compared with HGF (open reading frame) published by NCBI. The cDNA was synthesized using the cDNA synthesis kit (Invitrogen, Life te, Grand Island, NY, USA) using random hexamers. Afterwards, full-length human *NK4* cDNA was amplified using specific forward 5′-CTA**GCTAGC**GCCACCATGTGGGTGAC-3′ (NheI) and reverse 5′-ACGC**GTCGAC**TCAGACTATTGTAGGTGT-3′ (SalI) primers. The forward primer included six nucleotides (underlined) for Kozak sequence site. The polymerase chain reaction (PCR) products were then cloned into pYr-adshuttle-6 vector according to the manufacturer’s protocol (Invitrogen, Life te, Grand Island, NY, USA) and used for transformation of competent Escherichia coli DH5α. Then, the recombinant bacteria were screened using LB agar medium containing 100 μg/mL kanamycin. The presence of the insert was confirmed by PCR and double digestion of NheI and SalI. Finally, the fidelity of the cloned sequence was confirmed by DNA sequencing. The correct clone was called pYr-adshuttle-6-*NK4*.

### 3.5. Construction of Recombinant Ad Vector DNA Containing Human *NK4*

The Adeno pAd/BL-DEST adenovirus expression vector was purchased from Invitrogen. The LR recombination reaction was carried out between the clone (pYr-adshuttle-6-*NK4*) and destination vector (pAd/BL-DEST) according to the manufacturer’s protocols (Invitrogen).

### 3.6. Producing Viral Stocks and Transducing MSCs

The HEK293 cells were transfected with PacI linearized recombinant adenoviruses. In brief, the recombinant adenoviruses containing the entire coding sequence of *hNK4* (pAd-*NK4*) were digested with PacI to expose the ITRs. Then, the HEK293 cells were cultured in a 60-mm plate and transfected with the linear pAd-*NK4* at a confluency of 70%. The transfection reaction was carried out using the lipo 2000 reagent as described by the manufacturer (Invitrogen, Life te, Grand Island, NY, USA). In order to harvest the viruses, the cells were lysed with three consecutive freeze-thaw cycles, and then the viruses were collected from supernatant. Next, the adenoviruses were amplified by infecting additional HEK293 cells with the crude viral lysate, and then subjected to viral titer determination by plaque assay on HEK293 cells. The recombinant adenovirus containing *NK4* DNA was called rAd-*NK4*/EGFP.

Subsequently, for transduction of MSCs with rAd-*NK4*/EGFP, 8 × 10^5^ cells/well were seeded in six-well plates in growth medium containing DMEM-LG (Gibco, Life te, Grand Island, NY, USA) and 10% FBS and incubated for 12 h. Virus stock was diluted with serum-free low glucose DMEM and serial dilution of viral stock was added to the cells. Following determination of the appropriate MOI, MSCs were infected with rAd-*NK4*/EGFP. The *NK4* transduced MSCs were analyzed by reverse transcription polymerase chain reaction (RT-PCR) and western blotting for overexpression of *NK4*.

### 3.7. TCID_50_

Viral yield was determined by the limiting dilution method, known as the determination of 50% tissue culture infective dose, described previously [46]. The data from three separate infection studies were expressed as plaque-forming units per milliliter.

### 3.8. 3-(4,5,-Dimethyl thiazolyl-2)-2,5-diphenyl tetrazolium bromide (MTT)

MSCs were harvested after transduced with rAd-*NK4*/EGFP for various MOIs (0–200 h). A total of 300 mL MTT reagent (Sigma, St. Louis, MO, USA) was added to each well 2 h prior to harvest. The supernatant was then removed and incubated with 400 mL dimethylsulfoxide (Sigma, St. Louis, MO, USA) for 10 min. Absorbance at 540 nm was recorded using an enzyme-linked immunosorbent assay-plate reader.

### 3.9. Western Blot Analysis

Cells were harvested, washed twice with PBS, and lysed with buffer (20 mM Tris (pH 7.5), 1 mM EDTA, 1 mM EGTA, 1% Triton X-100, 1 mg/mL aprotinin, and 1 mM phenylmethylsulfonylfluoride (PMSF)) for 30 min on ice. The lysates were then cleared by centrifugation (22,250× *g* at 4 °C for 10 min). Protein concentration was determined by the Bradford method. Equal amounts of protein (20 mg) were resolved by 10% SDS-PAGE and transferred to a polyvinylidene fluoride (PVDF) membrane. After the blots were washed with TBST (tris-buffer solution-Tween-20) (10 mM Tris-HCl (pH 7.6), 150 mM NaCl, and 0.05% Tween-20), the membranes were blocked with 5% skim milk for 1 h and incubated with an appropriate primary antibody at the dilution recommended by the supplier. The membrane was then washed and primary antibodies were detected with a horseradish peroxidase-conjugated secondary antibody. The bands were visualized by enhanced chemiluminescence (Amersham Pharmacia Biotech Inc., GE, Fairfield, CT, USA). Densitometric analysis was performed using the TINA version 2.09 program package (DesignSoft, Budapest, Hungary). The ratios between treated and control samples were calculated for each individual experiment and expressed as a percentage of controls.

### 3.10. RT-PCR

Molecular characterization of cells used in differentiation assays was done by RT-PCR. Total RNA was extracted with the GenElute™ Mammalian Total RNA Miniprep Kit (Sigma-Aldrich, St. Louis, MO, USA). First-strand cDNA synthesis was performed on 1 μg of total RNA; cDNA samples were thereafter amplified in a thermocycler (Bio-Rad Laboratories, Hercules, CA, USA) for 35 cycles (94 °C for 45 s, 60 °C for 45 s and 72 °C for 45 s) with specific oligonucleotide primers (Metabion, Martinsried, Germany) to assess mRNA expression for *NK4* and *GAPDH*, as endogenous control. Enzymatically dissociated, freshly isolated MSC cells were used as controls. The oligonucleotide primer sequences for both RT-PCR are shown in Table 1.

### 3.11. Determination of MSC Supernatant NK4 with Enzyme-Linked Immunosorbent Assay

MSC supernatant was obtained from each treatment group. ELISA was performed by using Quantikine immunoassay from R & D Systems (Madison, WI, USA). In brief, supernantants were incubated in the precoated 96-well plates for 4 h at room temperature. After three washes, conjugated antibody was added for 2 h at room temperature, incubated in the substrate solution for 30 min, and the reaction was stopped by the addition of stop solution. The optical density of each well was determined by using a microplate reader at 450 nm.

### 3.12. Co-Culture Migration Assay

Migration assays were performed in transwell inserts with 8-μm pore uncoated membrane filters (Corning Incorporated, Corning, NY, USA). Pancreatic cancer cell SW1990, pancreatic epithelial cells and negative control were seeded onto the bottom chambers. MSCs were added to the upper chambers 24 h later. MSCs were allowed to migrate for 24 h at 37 °C. Then, the upper sides of the filters were carefully washed with cold PBS and non-migrated cells at the top of the filter were removed using a cotton swab. Cells that had migrated to the bottom of the filters were fixed with paraformaldehyde and crystal violet stained, and imaged with a Nikon Eclipse TE2000-U inverted epifluorescence microscope (Nikon, Tokyo, Japan). Stained cells were counted in 10 pictures taken at ×400 magnification. The average cell number counted per field was multiplied by the ratio between the surfaces (total/counted) to determine the total number of migrated cells. Inhibition of migration was calculated normalizing data to control cells.

### 3.13. Colony-Forming Assay

The MSCs and SW1990 were co-cultured as described above and plated in triplicate at 200 cells per well in 24-well plates. From the third day after plating, SW1990 cells were cultured with 1640 Medium (MEM) (Gibco, Invitrogen, Grand Island, NY, USA) with 10% FBS and antibiotics. Medium was changed three times a week and cells cultured at 37 °C in a humidified 5% CO_2_ incubator. Three weeks after seeding, cells were fixed with methanol for 15 min and stained with 5% Giemsa (Sigma-Aldrich, St. Louis, MO, USA). Colonies containing more than 10 cells were counted and their number was normalized to the inoculation cell number per well.

### 3.14. Cell Scatter Assay

SW1990 cells (2.5 × 10^3^ cells/well) were seeded into 12-well plates. After culturing for 48 h at 37 °C, the medium was replaced with 2% cell maintenance medium. The cell cultures were divided into a control group, a rAd-EGFP-MSC group (100 MOI), and a rAd-*NK4*/EGFP-MSC group 100 MOI) and treated with recombinant human HGF (PeproTech EC, Rocky Hill, NJ, USA) at a final concentration of 10 μg/L for 48 h. At the end of the experiment, cells were fixed with formalin and stained with crystal violet. The cell colony scattering was observed and photographed.

### 3.15. Cell Cycle Detection

The MSCs and SW1990 were co-cultured at 2 × 10^5^ cells/well. Cells were obtained after 72 h, and adherent cells were digested by trypsin, washed with PBS and fixed with 70% pre-cooled ethanol at 4 °C overnight. An equal amount of PBS was added twice for washing. Up to 5 μL RNase A was added at 37 °C for 1 h, followed by the addition of propidium iodide at 4 °C in the dark for 30 min. Cell cycle was analyzed by flow cytometry using the MCYCLE software (version 1.0, Beckman, New York, NY, USA).

### 3.16. Statistical Analysis

All the images shown in this paper are representative of at least three independent experiments carried out under the same conditions. Statistical analysis was performed by ANOVA test followed by Bonferroni’s multiple comparison tests. *p* < 0.05 were considered statistically significant. Data are expressed as the mean ± standard deviation (SD).

## 4. Conclusions

In conclusion, we demonstrated that *NK4*-MSCs inhibited the pancreatic cancer progression *in vitro*. Proliferation of pancreatic cancer cells was inhibited by interfering with EMT and the cell cycle. This *NK4*-MSC-based therapy could therefore be an attractive drug-delivery system for homing in and then attacking the cancer. This approach would be not only a powerful but also a safe strategy for targeting the milieu of malignant cells.

## Figures and Tables

**Table 1.** Primer for RT-PCR.

Gene name	Primer	5′-3′
*NK4*	Sense	ATCCAAGGTCAAGGAGAAGGCT
	Anti-sense	TTCACAACGAGAAATAGGGCA

*GAPDH*	Sense	ACCACAGTCCATGCCATCAC
	Anti-sense	TCCACCACCCTGTTGCTGTA
